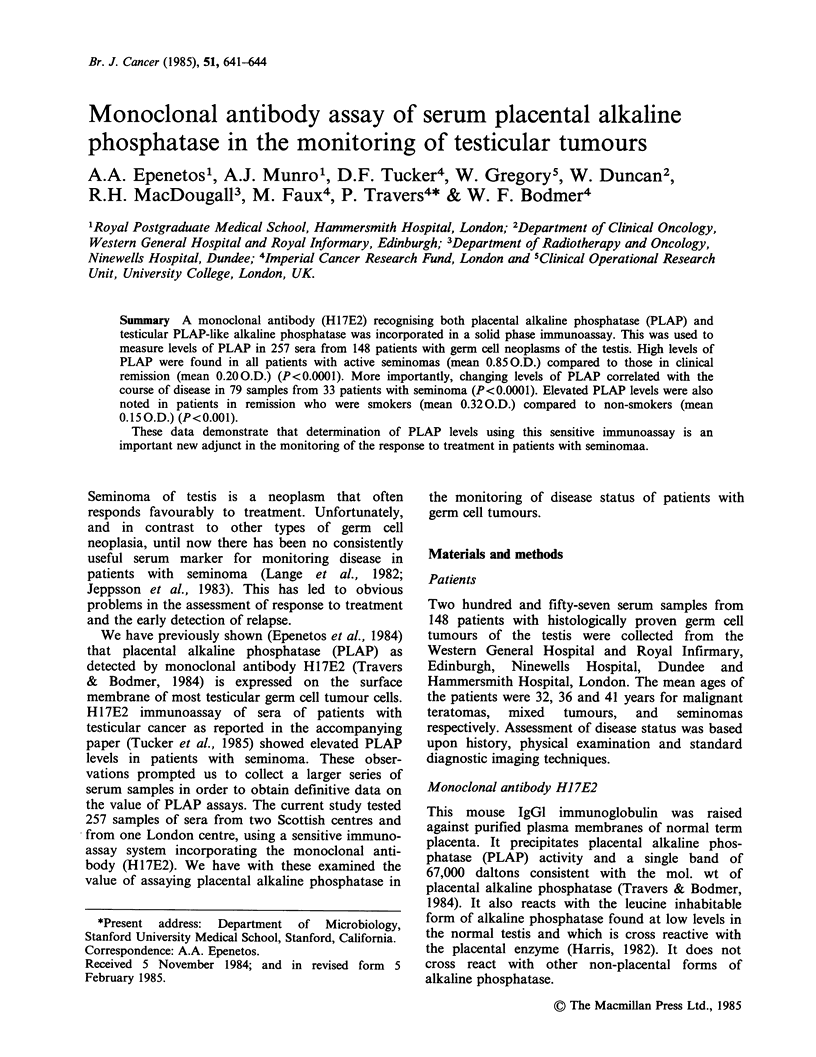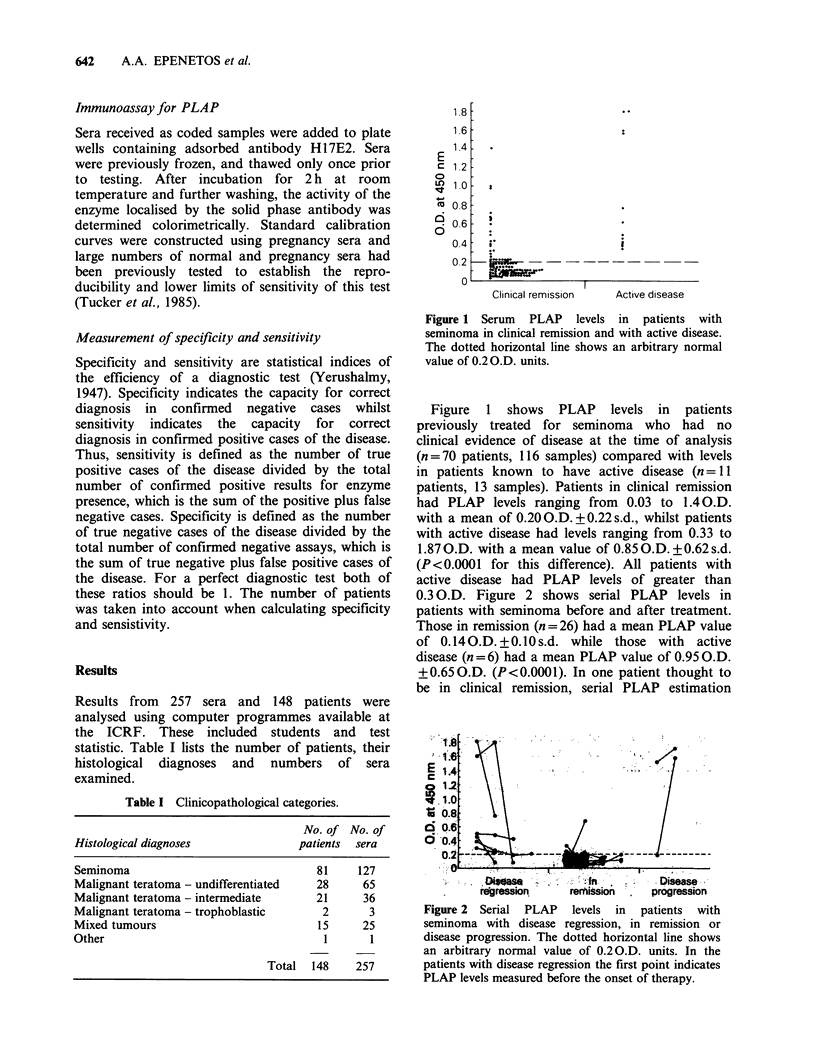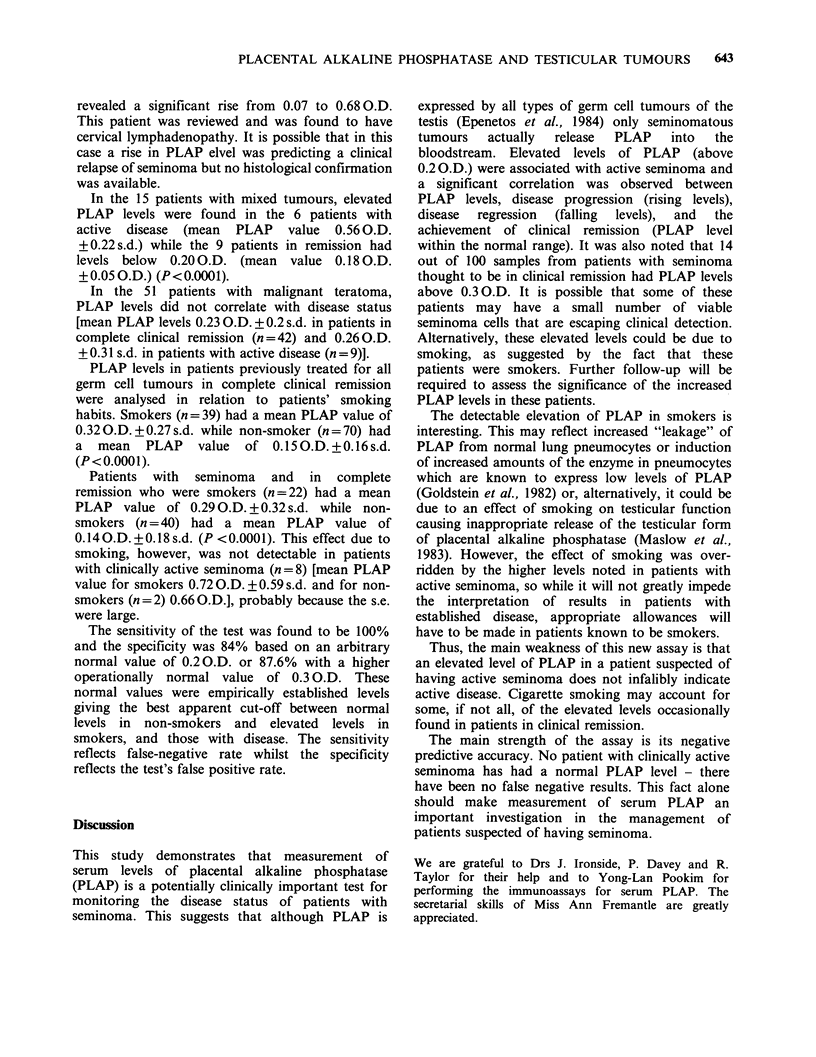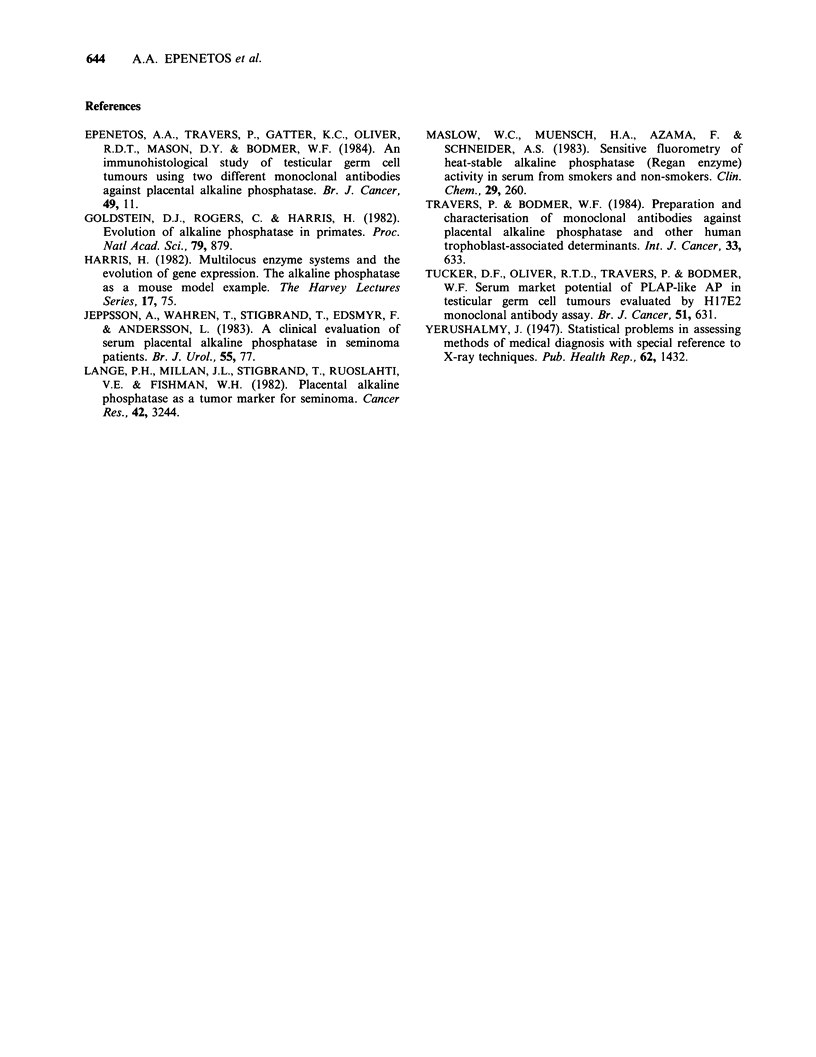# Monoclonal antibody assay of serum placental alkaline phosphatase in the monitoring of testicular tumours.

**DOI:** 10.1038/bjc.1985.96

**Published:** 1985-05

**Authors:** A. A. Epenetos, A. J. Munro, D. F. Tucker, W. Gregory, W. Duncan, R. H. MacDougall, M. Faux, P. Travers, W. F. Bodmer

## Abstract

A monoclonal antibody (H17E2) recognising both placental alkaline phosphatase (PLAP) and testicular PLAP-like alkaline phosphatase was incorporated in a solid phase immunoassay. This was used to measure levels of PLAP in 257 sera from 148 patients with germ cell neoplasms of the testis. High levels of PLAP were found in all patients with active seminomas (mean 0.85 O.D.) compared to those in clinical remission (mean 0.20 O.D.) (P less than 0.0001). More importantly, changing levels of PLAP correlated with the course of disease in 79 samples from 33 patients with seminoma (P less than 0.0001). Elevated PLAP levels were also noted in patients in remission who were smokers (mean 0.32 O.D.) compared to non-smokers (mean 0.15 O.D.) (P less than 0.001). These data demonstrate that determination of PLAP levels using this sensitive immunoassay is an important new adjunct in the monitoring of the response to treatment in patients with seminoma.


Br. J. Cancer (1985), 51, 641–644

### Monoclonal antibody assay of serum placental alkaline phosphatase in the monitoring of testicular tumours

A.A. Epenetos[1], A.J. Munro[1], D.F. Tucker[4], W. Gregory[5], W. Duncan[2], R.H. MacDougall[3], M. Faux[4], P. Travers[4]* & W. F. Bodmer[4]

[1]*Royal Postgraduate Medical School, Hammersmith Hospital, London;* [2]*Department of Clinical Oncology, Western General Hospital and Royal Informary, Edinburgh;* [3]*Department of Radiotherapy and Oncology, Ninewells Hospital, Dundee;* [4]*Imperial Cancer Research Fund, London and* [5]*Clinical Operational Research Unit, University College, London, UK.*

**Summary** A monoclonal antibody (H17E2) recognising both placental alkaline phosphatase (PLAP) and testicular PLAP-like alkaline phosphatase was incorporated in a solid phase immunoassay. This was used to measure levels of PLAP in 257 sera from 148 patients with germ cell neoplasms of the testis. High levels of PLAP were found in all patients with active seminomas (mean 0.85 O.D.) compared to those in clinical remission (mean 0.20 O.D.) ($P<0.0001$). More importantly, changing levels of PLAP correlated with the course of disease in 79 samples from 33 patients with seminoma ($P<0.0001$). Elevated PLAP levels were also noted in patients in remission who were smokers (mean 0.32 O.D.) compared to non-smokers (mean 0.15 O.D.) ($P<0.001$).

These data demonstrate that determination of PLAP levels using this sensitive immunoassay is an important new adjunct in the monitoring of the response to treatment in patients with seminomaa.

Seminoma of testis is a neoplasm that often responds favourably to treatment. Unfortunately, and in contrast to other types of germ cell neoplasia, until now there has been no consistently useful serum marker for monitoring disease in patients with seminoma (Lange *et al.*, 1982; Jeppsson *et al.*, 1983). This has led to obvious problems in the assessment of response to treatment and the early detection of relapse.

We have previously shown (Epenetos *et al.*, 1984) that placental alkaline phosphatase (PLAP) as detected by monoclonal antibody H17E2 (Travers & Bodmer, 1984) is expressed on the surface membrane of most testicular germ cell tumour cells. H17E2 immunoassay of sera of patients with testicular cancer as reported in the accompanying paper (Tucker *et al.*, 1985) showed elevated PLAP levels in patients with seminoma. These observations prompted us to collect a larger series of serum samples in order to obtain definitive data on the value of PLAP assays. The current study tested 257 samples of sera from two Scottish centres and from one London centre, using a sensitive immunoassay system incorporating the monoclonal antibody (H17E2). We have with these examined the value of assaying placental alkaline phosphatase in the monitoring of disease status of patients with germ cell tumours.

*Present address: Department of Microbiology, Stanford University Medical School, Stanford, California.
Correspondence: A.A. Epenetos.
Received 5 November 1984; and in revised form 5 February 1985.

### Materials and methods

### *Patients*

Two hundred and fifty-seven serum samples from 148 patients with histologically proven germ cell tumours of the testis were collected from the Western General Hospital and Royal Infirmary, Edinburgh, Ninewells Hospital, Dundee and Hammersmith Hospital, London. The mean ages of the patients were 32, 36 and 41 years for malignant teratomas, mixed tumours, and seminomas respectively. Assessment of disease status was based upon history, physical examination and standard diagnostic imaging techniques.

### *Monoclonal antibody H17E2*

This mouse IgGl immunoglobulin was raised against purified plasma membranes of normal term placenta. It precipitates placental alkaline phosphatase (PLAP) activity and a single band of 67,000 daltons consistent with the mol. wt of placental alkaline phosphatase (Travers & Bodmer, 1984). It also reacts with the leucine inhabitable form of alkaline phosphatase found at low levels in the normal testis and which is cross reactive with the placental enzyme (Harris, 1982). It does not cross react with other non-placental forms of alkaline phosphatase.

© The Macmillan Press Ltd., 1985

*Immunoassay for PLAP*

Sera received as coded samples were added to plate wells containing adsorbed antibody H17E2. Sera were previously frozen, and thawed only once prior to testing. After incubation for 2 h at room temperature and further washing, the activity of the enzyme localised by the solid phase antibody was determined colorimetrically. Standard calibration curves were constructed using pregnancy sera and large numbers of normal and pregnancy sera had been previously tested to establish the reproducibility and lower limits of sensitivity of this test (Tucker *et al.*, 1985).

*Measurement of specificity and sensitivity*

Specificity and sensitivity are statistical indices of the efficiency of a diagnostic test (Yerushalmy, 1947). Specificity indicates the capacity for correct diagnosis in confirmed negative cases whilst sensitivity indicates the capacity for correct diagnosis in confirmed positive cases of the disease. Thus, sensitivity is defined as the number of true positive cases of the disease divided by the total number of confirmed positive results for enzyme presence, which is the sum of the positive plus false negative cases. Specificity is defined as the number of true negative cases of the disease divided by the total number of confirmed negative assays, which is the sum of true negative plus false positive cases of the disease. For a perfect diagnostic test both of these ratios should be 1. The number of patients was taken into account when calculating specificity and sensistivity.

**Results**

Results from 257 sera and 148 patients were analysed using computer programmes available at the ICRF. These included students and test statistic. Table I lists the number of patients, their histological diagnoses and numbers of sera examined.

**Table I** Clinicopathological categories.

| *Histological diagnoses* | *No. of patients* | *No. of sera* |
|---|---|---|
| Seminoma | 81 | 127 |
| Malignant teratoma – undifferentiated | 28 | 65 |
| Malignant teratoma – intermediate | 21 | 36 |
| Malignant teratoma – trophoblastic | 2 | 3 |
| Mixed tumours | 15 | 25 |
| Other | 1 | 1 |
| Total | 148 | 257 |

**Figure 1** Serum PLAP levels in patients with seminoma in clinical remission and with active disease. The dotted horizontal line shows an arbitrary normal value of 0.2 O.D. units.

Figure 1 shows PLAP levels in patients previously treated for seminoma who had no clinical evidence of disease at the time of analysis ($n=70$ patients, 116 samples) compared with levels in patients known to have active disease ($n=11$ patients, 13 samples). Patients in clinical remission had PLAP levels ranging from 0.03 to 1.4 O.D. with a mean of 0.20 O.D. $\pm$0.22 s.d., whilst patients with active disease had levels ranging from 0.33 to 1.87 O.D. with a mean value of 0.85 O.D. $\pm$0.62 s.d. ($P<0.0001$ for this difference). All patients with active disease had PLAP levels of greater than 0.3 O.D. Figure 2 shows serial PLAP levels in patients with seminoma before and after treatment. Those in remission ($n=26$) had a mean PLAP value of 0.14 O.D. $\pm$0.10 s.d. while those with active disease ($n=6$) had a mean PLAP value of 0.95 O.D. $\pm$0.65 O.D. ($P<0.0001$). In one patient thought to be in clinical remission, serial PLAP estimation

**Figure 2** Serial PLAP levels in patients with seminoma with disease regression, in remission or disease progression. The dotted horizontal line shows an arbitrary normal value of 0.2 O.D. units. In the patients with disease regression the first point indicates PLAP levels measured before the onset of therapy.

revealed a significant rise from 0.07 to 0.68 O.D. This patient was reviewed and was found to have cervical lymphadenopathy. It is possible that in this case a rise in PLAP elvel was predicting a clinical relapse of seminoma but no histological confirmation was available.

In the 15 patients with mixed tumours, elevated PLAP levels were found in the 6 patients with active disease (mean PLAP value 0.56 O.D. ±0.22 s.d.) while the 9 patients in remission had levels below 0.20 O.D. (mean value 0.18 O.D. ±0.05 O.D.) ($P<0.0001$).

In the 51 patients with malignant teratoma, PLAP levels did not correlate with disease status [mean PLAP levels 0.23 O.D. ±0.2 s.d. in patients in complete clinical remission ($n=42$) and 0.26 O.D. ±0.31 s.d. in patients with active disease ($n=9$)].

PLAP levels in patients previously treated for all germ cell tumours in complete clinical remission were analysed in relation to patients' smoking habits. Smokers ($n=39$) had a mean PLAP value of 0.32 O.D. ±0.27 s.d. while non-smoker ($n=70$) had a mean PLAP value of 0.15 O.D. ±0.16 s.d. ($P<0.0001$).

Patients with seminoma and in complete remission who were smokers ($n=22$) had a mean PLAP value of 0.29 O.D. ±0.32 s.d. while non-smokers ($n=40$) had a mean PLAP value of 0.14 O.D. ±0.18 s.d. ($P<0.0001$). This effect due to smoking, however, was not detectable in patients with clinically active seminoma ($n=8$) [mean PLAP value for smokers 0.72 O.D. ±0.59 s.d. and for non-smokers ($n=2$) 0.66 O.D.], probably because the s.e. were large.

The sensitivity of the test was found to be 100% and the specificity was 84% based on an arbitrary normal value of 0.2 O.D. or 87.6% with a higher operationally normal value of 0.3 O.D. These normal values were empirically established levels giving the best apparent cut-off between normal levels in non-smokers and elevated levels in smokers, and those with disease. The sensitivity reflects false-negative rate whilst the specificity reflects the test's false positive rate.

### Discussion

This study demonstrates that measurement of serum levels of placental alkaline phosphatase (PLAP) is a potentially clinically important test for monitoring the disease status of patients with seminoma. This suggests that although PLAP is expressed by all types of germ cell tumours of the testis (Epenetos *et al.*, 1984) only seminomatous tumours actually release PLAP into the bloodstream. Elevated levels of PLAP (above 0.2 O.D.) were associated with active seminoma and a significant correlation was observed between PLAP levels, disease progression (rising levels), disease regression (falling levels), and the achievement of clinical remission (PLAP level within the normal range). It was also noted that 14 out of 100 samples from patients with seminoma thought to be in clinical remission had PLAP levels above 0.3 O.D. It is possible that some of these patients may have a small number of viable seminoma cells that are escaping clinical detection. Alternatively, these elevated levels could be due to smoking, as suggested by the fact that these patients were smokers. Further follow-up will be required to assess the significance of the increased PLAP levels in these patients.

The detectable elevation of PLAP in smokers is interesting. This may reflect increased "leakage" of PLAP from normal lung pneumocytes or induction of increased amounts of the enzyme in pneumocytes which are known to express low levels of PLAP (Goldstein *et al.*, 1982) or, alternatively, it could be due to an effect of smoking on testicular function causing inappropriate release of the testicular form of placental alkaline phosphatase (Maslow *et al.*, 1983). However, the effect of smoking was over-ridden by the higher levels noted in patients with active seminoma, so while it will not greatly impede the interpretation of results in patients with established disease, appropriate allowances will have to be made in patients known to be smokers.

Thus, the main weakness of this new assay is that an elevated level of PLAP in a patient suspected of having active seminoma does not infalibly indicate active disease. Cigarette smoking may account for some, if not all, of the elevated levels occasionally found in patients in clinical remission.

The main strength of the assay is its negative predictive accuracy. No patient with clinically active seminoma has had a normal PLAP level – there have been no false negative results. This fact alone should make measurement of serum PLAP an important investigation in the management of patients suspected of having seminoma.

We are grateful to Drs J. Ironside, P. Davey and R. Taylor for their help and to Yong-Lan Pookim for performing the immunoassays for serum PLAP. The secretarial skills of Miss Ann Fremantle are greatly appreciated.

### References

EPENETOS, A.A., TRAVERS, P., GATTER, K.C., OLIVER, R.D.T., MASON, D.Y. & BODMER, W.F. (1984). An immunohistological study of testicular germ cell tumours using two different monoclonal antibodies against placental alkaline phosphatase. *Br. J. Cancer*, **49,** 11.

GOLDSTEIN, D.J., ROGERS, C. & HARRIS, H. (1982). Evolution of alkaline phosphatase in primates. *Proc. Natl Acad. Sci.*, **79,** 879.

HARRIS, H. (1982). Multilocus enzyme systems and the evolution of gene expression. The alkaline phosphatase as a mouse model example. *The Harvey Lectures Series*, **17,** 75.

JEPPSSON, A., WAHREN, T., STIGBRAND, T., EDSMYR, F. & ANDERSSON, L. (1983). A clinical evaluation of serum placental alkaline phosphatase in seminoma patients. *Br. J. Urol.*, **55,** 77.

LANGE, P.H., MILLAN, J.L., STIGBRAND, T., RUOSLAHTI, V.E. & FISHMAN, W.H. (1982). Placental alkaline phosphatase as a tumor marker for seminoma. *Cancer Res.*, **42,** 3244.

MASLOW, W.C., MUENSCH, H.A., AZAMA, F. & SCHNEIDER, A.S. (1983). Sensitive fluorometry of heat-stable alkaline phosphatase (Regan enzyme) activity in serum from smokers and non-smokers. *Clin. Chem.*, **29,** 260.

TRAVERS, P. & BODMER, W.F. (1984). Preparation and characterisation of monoclonal antibodies against placental alkaline phosphatase and other human trophoblast-associated determinants. *Int. J. Cancer*, **33,** 633.

TUCKER, D.F., OLIVER, R.T.D., TRAVERS, P. & BODMER, W.F. Serum market potential of PLAP-like AP in testicular germ cell tumours evaluated by H17E2 monoclonal antibody assay. *Br. J. Cancer*, **51,** 631.

YERUSHALMY, J. (1947). Statistical problems in assessing methods of medical diagnosis with special reference to X-ray techniques. *Pub. Health Rep.*, **62,** 1432.